# Myocardial ^18^F-FDG Uptake Pattern for Cardiovascular Risk Stratification in Patients Undergoing Oncologic PET/CT

**DOI:** 10.3390/jcm9072279

**Published:** 2020-07-17

**Authors:** Achi Haider, Susan Bengs, Katharina Schade, Winandus J. Wijnen, Angela Portmann, Dominik Etter, Sandro Fröhlich, Geoffrey I. Warnock, Valerie Treyer, Irene A. Burger, Michael Fiechter, Ken Kudura, Tobias A. Fuchs, Aju P. Pazhenkottil, Ronny R. Buechel, Philipp A. Kaufmann, Alexander Meisel, Paul Stolzmann, Catherine Gebhard

**Affiliations:** 1 Department of Nuclear Medicine, University Hospital Zurich, 8091 Zurich, Switzerland; susan.bengs@usz.ch (S.B.); katharinaalexandra.schade@usz.ch (K.S.); Winandus.Wijnen@usz.ch (W.J.W.); angela.portmann@usz.ch (A.P.); dominik.etter@usz.ch (D.E.); Sandro.Froehlich@usz.ch (S.F.); geoffreyiain.warnock@uzh.ch (G.I.W.); valerie.treyer@usz.ch (V.T.); irene.burger@usz.ch (I.A.B.); michael.fiechter@usz.ch (M.F.); ken.kudura@usz.ch (K.K.); tobias.fuchs@usz.ch (T.A.F.); aju.pazhenkottil@usz.ch (A.P.P.); ronny.buechel@usz.ch (R.R.B.); pak@usz.ch (P.A.K.); alexander.meisel@usz.ch (A.M.); paul.stolzmann@usz.ch (P.S.); catherine.gebhard@usz.ch (C.G.); 2 Center for Molecular Cardiology, University of Zurich, 8952 Schlieren, Switzerland; 3 Swiss Paraplegic Center, 6207 Nottwil, Switzerland; 4 Department of Internal Medicine II, Division of Cardiology, Medical University of Vienna, 1090 Vienna, Austria

**Keywords:** myocardial ^18^F-FDG uptake pattern, oncologic PET/CT, cardiovascular risk, heart failure, cardio-oncology

## Abstract

Objective: Positron emission tomography/computed tomography with ^18^F-fluorodeoxy-glucose (^18^F-FDG-PET/CT) has become the standard staging modality in various tumor entities. Cancer patients frequently receive cardio-toxic therapies. However, routine cardiovascular assessment in oncologic patients is not performed in current clinical practice. Accordingly, this study sought to assess whether myocardial ^18^F-FDG uptake patterns of patients undergoing oncologic PET/CT can be used for cardiovascular risk stratification. Methods: Myocardial ^18^F-FDG uptake pattern was assessed in 302 patients undergoing both oncologic whole-body ^18^F-FDG-PET/CT and myocardial perfusion imaging by single-photon emission computed tomography (SPECT-MPI) within a six-month period. Primary outcomes were myocardial ^18^F-FDG uptake pattern, impaired myocardial perfusion, ongoing ischemia, myocardial scar, and left ventricular ejection fraction. Results: Among all patients, 109 (36.1%) displayed no myocardial ^18^F-FDG uptake, 77 (25.5%) showed diffuse myocardial ^18^F-FDG uptake, 24 (7.9%) showed focal ^18^F-FDG uptake, and 92 (30.5%) had a focal on diffuse myocardial ^18^F-FDG uptake pattern. In contrast to the other uptake patterns, focal myocardial ^18^F-FDG uptake was predominantly observed in patients with myocardial abnormalities (i.e., abnormal perfusion, impaired LVEF, myocardial ischemia, or scar). Accordingly, a multivariate logistic regression identified focal myocardial ^18^F-FDG uptake as a strong predictor of abnormal myocardial function/perfusion (odds ratio (OR) 5.32, 95% confidence interval (CI) 1.73–16.34, *p* = 0.003). Similarly, focal myocardial ^18^F-FDG uptake was an independent predictor of ongoing ischemia and myocardial scar (OR 4.17, 95% CI 1.53–11.4, *p* = 0.005 and OR 3.78, 95% CI 1.47–9.69, *p* = 0.006, respectively). Conclusions: Focal myocardial ^18^F-FDG uptake seen on oncologic PET/CT indicates a significantly increased risk for multiple myocardial abnormalities. Obtaining and taking this information into account will help to stratify patients according to risk and will reduce unnecessary cardiovascular complications in cancer patients.

## 1. Introduction

^18^F-fluorodeoxyglucose positron emission tomography/computed tomography (^18^F-FDG-PET/CT) is widely used for initial cancer staging [1,2]. It provides incremental prognostic information and enables personalized anti-neoplastic therapy [3,4]. Consequently, ^18^F-FDG-PET/CT is considered the standard of care for various tumor entities, as it improves cancer outcomes and reduces unnecessary surgeries and thus complications [5].

In addition to its established role in oncology, PET/CT imaging has significantly improved clinical decision-making in patients with known or suspected coronary artery disease (CAD) [6,7,8,9,10,11]. Although cardiovascular and oncologic ^18^F-FDG-PET/CT imaging procedures are similar, there are critical differences in patient preparation protocols. While cardiovascular ^18^F-FDG-PET/CT has been validated to assess myocardial viability and cardiac inflammation, it remains unknown whether oncologic ^18^F-FDG-PET/CT can identify patients at risk for cardiovascular disease [12,13,14,15]. However, a timely and cost-effective risk stratification of cancer patients is crucial given that cardiotoxicity represents the most critical complication of anti-cancer treatments [16]. Indeed, a recent population-based study encompassing more than 3 million US cancer patients revealed that the highest number of cardiovascular deaths occurred in the first year following initial cancer diagnosis [17]. This finding was attributed to the aggressive treatment at initial cancer diagnosis and calls for a close cardiovascular monitoring; however, routine cardiovascular assessment in oncologic patients is not yet performed in current clinical practice.

Given the routine use of ^18^F-FDG-PET/CT in oncology, identifying a high-risk ^18^F-FDG-PET/CT myocardial uptake pattern would allow for the referral of vulnerable patients to further cardiovascular assessment. Thus, we investigated the value of myocardial ^18^F-FDG uptake pattern to predict cardiovascular risk in patients undergoing oncologic ^18^F-FDG-PET/CT.

## 2. Materials and Methods

### 2.1. Study Population

Our study population was selected from a retrospective cohort study of consecutive patients undergoing whole-body ^18^F-FDG-PET/CT for malignant disorders at the University Hospital Zurich between November 2007 and February 2015. Out of 25,600 cases, 10,148 patients underwent a 1-day stress/rest (adenosine, dobutamine, or exercise) myocardial perfusion imaging by ^99m^Tc-tetrofosmin single-photon emission computed tomography (SPECT-MPI) including non-contrast-enhanced CT for attenuation correction to evaluate known or suspected CAD. A total of 332 study patients underwent both whole-body ^18^F-FDG-PET/CT and SPECT-MPI within a 6-month period. A total of 24 patients were excluded due to insufficient image quality and/or clinical data. Hence, the final study population consisted of 302 patients. Demographic parameters and key factors of the patients’ histories were obtained by review of medical records. This study was approved by the local ethics committee (BASEC No. 2017-01112) and the need for informed consent was waived due to the retrospective nature of the study. The study population was shared with another registry [18].

### 2.2. 99 ^m^Tc-Tetrofosmin SPECT-MPI

Patients underwent an electrocardiography (ECG)-gated 1-day stress/rest protocol as previously described [19]. We acquired SPECT-MPI with a dual-head camera (Infinia Hawkeye, Ventri) or with a Discovery NM/CT 530c/570c (GE Healthcare, Milwaukee, WI, USA). Cedars QGS/QPS software (Cedars-Sinai Medical Center, Los Angeles, CA, USA) was used to evaluate SPECT-MPI in accordance with current guidelines [20,21]. Each myocardial segment was scored by applying a 20-segment model and 5-point scoring system (consensus of two experts) as previously reported [19,20,21,22]: 0 = normal, 1 = equivocal, 2 = moderate, 3 = severe reduction in tracer uptake, and 4 = absence of detectable radioligand. Stress scores ≥2 in two or more segments were considered abnormal. Mismatch between stress and rest scores with a rest score ≤1 or a stress score of 4 combined with a rest score of 2 were considered a reversible perfusion defect (i.e., ischemia). Left ventricular (LV) volumes were derived from gated SPECT images, and the left ventricular ejection fraction (LVEF) was calculated by dividing stroke volume (end-diastolic volume (EDV)—end-systolic volume (ESV)) by EDV. For coronary calcium scoring (CACS) and attenuation correction, a non-contrast CT exam was conducted using a 64-slice CT scanner (LightSpeed VCT (2007–2010) or Slice Discovery HD 750 (2010–2014) or Revolution CT (2015), GE Healthcare) with the following parameters: 64 × 2.5 mm collimation, rotation time of 0.35 s, tube voltage of 120 kV, and tube current of 200 mA [23]. CACS (Agatston units, AU) was quantified with the semi-automatic SmartScore software (GE Healthcare, Milwaukee, WI, USA). Segments with coronary artery stent implantation or bypass-vessels were excluded from CACS assessment.

### 2.3. Whole-Body ^18^F-FDG PET/CT and Assessment of Myocardial ^18^F-FDG Uptake

Patients were instructed to fast for at least 4 h before administrating ^18^F-FDG. After measuring blood glucose, ^18^F-FDG was injected (334.5 MBq, range 180-409 MBq) into a peripheral vein. One hour later, patients underwent PET/CT imaging from skull to pelvis (including a non-contrast CT scan). Images were acquired in 3D mode on a Discovery VCT or Discovery RX scanner (GE-Healthcare, Milwaukee, WI, USA). PET/CT and CT images were merged and analyzed using Advantage Window Volume Viewer software (GE-Healthcare, Milwaukee, WI, USA).

Quantitative myocardial ^18^F-FDG uptake was measured by serially placing volumes of interest (VOI) using AW Server software (version 3.2, GE Healthcare) as previously described and validated [24,25,26,27,28]. VOIs were placed as follows: VOI 1 was placed at the mid-ventricular septum, VOI 2 at the anterior apex, VOI 3 at the mid-ventricular lateral wall, VOI 4 basal anterior wall, VOI 5 at the posterior apex, VOI 6 at the liver in an area of low ^18^F-FDG uptake, and VOI 7 at the aortic arch. For each VOI, we quantified VOI_size_ (volume of the volumetric region of interest in mm^3^), SUV_max_ (maximum standardized uptake value of ^18^F-FDG within any voxel of the VOI), SUV_av_ (average standardized uptake value of the tracer in all voxels of the VOI) and Vol, which is a threshold-dependent version of VOI_size_. ^18^F-FDG uptake pattern in the myocardium were assessed at a contrast level ranging between 0 and 10 g/mL.

**Figure 1:**
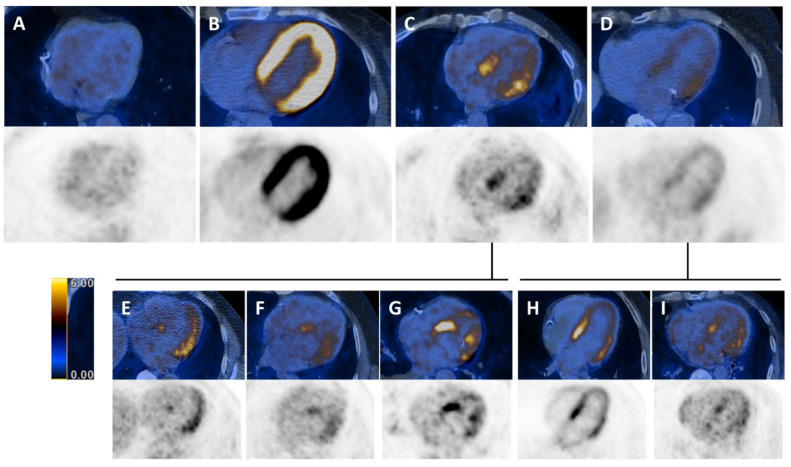
Classification of ^18^F-fluorodeoxy-glucose (^18^F-FDG) uptake patterns in the left ventricular wall. Positron emission tomography (PET) images superimposed on computed tomography (CT) images (colored) and PET images alone (black and white). (**A**) No myocardial ^18^F-FDG uptake. (**B**) Diffuse myocardial ^18^F-FDG uptake. (**C**) Focal myocardial ^18^F-FDG uptake. (**D**) Focal on diffuse myocardial ^18^F-FDG uptake. (**E**) Focal over half. (**F**) Focal spot. (**G**) Focal ring. (**H**) Focal on diffuse over half. (**I**) Focal on diffuse spot.

As described by Nose et al. [29], myocardial ^18^F-FDG uptake pattern can be classified as follows: (1) None (no myocardial ^18^F-FDG uptake), (2) diffuse (diffuse and homogeneous ^18^F-FDG uptake in the left ventricle), (3) focal myocardial ^18^F-FDG uptake, and (4) focal on diffuse (focal ^18^F-FDG uptake overlying the diffuse pattern) (Figure 1A–D). The ^18^F-FDG uptake pattern (3) focal and (4) focal on diffuse were further subdivided according to additional characteristics such as *ring pattern* (diffuse accumulation of ^18^F-FDG in the basal left ventricular wall), *over half pattern* (more than 50% accumulation), and *spotted pattern* (less than 50% accumulation of ^18^F-FDG) (Figure 1E–I). ^18^F-FDG uptake pattern were determined at three levels of the left ventricle: Basal, mid-ventricular, and apical. Right ventricular uptake patterns were not analyzed in the present study.

### 2.4. Statistical Analysis

Baseline characteristics were reported as frequencies with percentages for categorical variables and mean ± standard deviation (SD) for continuous variables. Prior to analysis, basic assumptions were confirmed by histogram plots. Hypotheses were tested with unpaired Student’s *t*-test, Mann–Whitney U test, analysis of variance (ANOVA), or Chi-square test, as appropriate. A multivariate logistic regression adjusted for age, sex, body mass index, injected ^18^F-FDG dose, and blood glucose level was applied to assess the predictive value of ^18^F-FDG myocardial uptake pattern for abnormal MPI findings, myocardial fibrosis, and left ventricular dysfunction. Statistical analyses were performed with IBM SPSS version 25.0 (SPSS Inc., Chicago, IL, USA). All tests were two-sided and a *p*-value of 0.05 was considered statistically significant.

## 3. Results

### 3.1. Study Population

We investigated the association of myocardial ^18^F-FDG uptake patterns with myocardial perfusion and the LVEF in 302 patients (70.9% men; mean age 66.8 ± 10.2 years). Reasons for referral for ^18^F-FDG PET were suspected or known malignancies including breast cancer, pulmonary cancer, colon cancer, cholangiocarcinoma, and esophageal cancer (Supplemental Figure S1). Of the patients, 109 (36.1%) had no myocardial ^18^F-FDG uptake, 77 (25.5%) had diffuse uptake, 24 (7.9%) had focal uptake, and in 92 (30.5%) a focal on diffuse myocardial uptake pattern was found (Supplementary Material, Table S1). Patients with focal myocardial ^18^F-FDG uptake had significantly higher *N*-terminal proB-type natriuretic peptide (NT proBNP) plasma levels than other groups (Table 1); otherwise, no significant differences were observed between groups in the prevalence of active malignancies and baseline characteristics (age, smoking, hypertension, diabetes, dyslipidemia, positive family history, known CAD, previous myocardial infarction (MI), previous percutaneous coronary intervention (PCI)/coronary artery bypass graft (CABG), clinical symptoms, and medical treatment) (Table 1). When baseline characteristics were stratified by myocardial perfusion/function findings, abnormal myocardial perfusion/function was more frequently observed in patients with known CAD, previous MI, and previous PCI/CABG (*p* < 0.001, Table 2). No significant differences were observed between patients with normal vs. abnormal perfusion/function for NT proBNP levels (*p* = 0.08, Table 2). Further, NT proBNP was not a significant predictor of abnormal perfusion/function at univariate analysis. Accordingly, it was not included in the multivariate regression models.

### 3.2. Association of ^18^F-FDG Uptake Patterns with Impaired Myocardial Function

**Figure 2:**
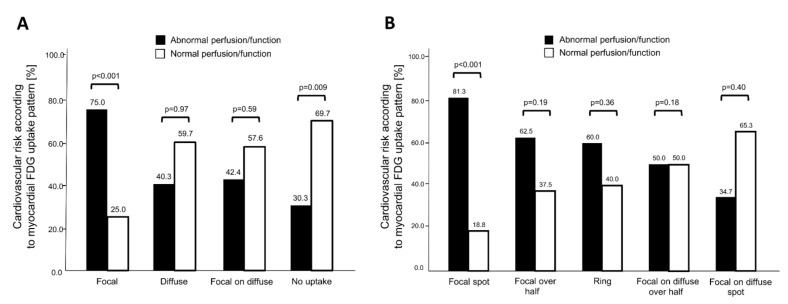
Cardiovascular risk according to myocardial uptake pattern of ^18^F-FDG-PET/CT. Patients were stratified by normal/abnormal myocardial perfusion imaging (MPI) findings. The Y-axis depicts the percentage of patients with abnormal and normal myocardial perfusion/function, respectively, in each subpopulation of distinct myocardial uptake pattern. The total number of patients within a subpopulation is set as 100%. (**A**) While focal myocardial ^18^F-FDG uptake was predominantly observed in patients with abnormal perfusion and function, patients with no myocardial ^18^F-FDG uptake more frequently presented with normal cardiac perfusion/function. (**B**) Dividing focal and focal on diffuse patterns into subgroups revealed that the focal spot pattern further improved risk prediction for cardiac abnormalities.

**Figure 3:**
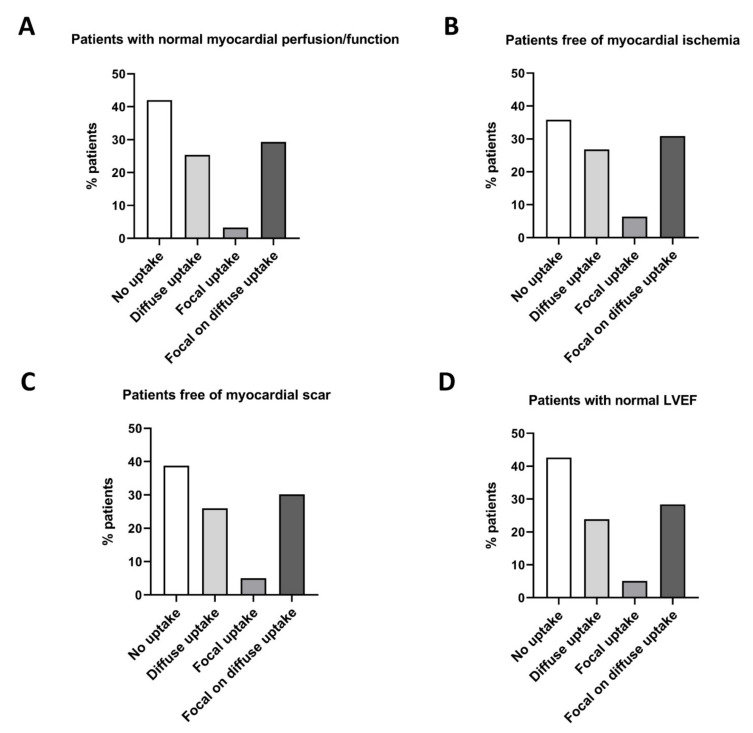
Left ventricular ^18^F-FDG-PET/CT uptake pattern in patients with normal myocardial perfusion imaging (MPI) findings. (**A**) Patients with normal myocardial perfusion/function. (**B**) Patients free of myocardial ischemia. (**C**) Patients free of myocardial scar. (**D**) Patients with a normal left ventricular ejection fraction (LVEF).

Seventy percent of patients with normal MPI findings had no myocardial ^18^F-FDG uptake, while the latter was found in only 30% of patients with abnormal MPI findings (*p* = 0.009, Figure 2A). In contrast, patients with focal myocardial ^18^F-FDG uptake more frequently had abnormal MPI (25.0% normal vs. 75.0% abnormal MPI, *p* < 0.001, Figure 2A). We found no significant differences between MPI findings in patients with diffuse or focal on diffuse myocardial ^18^F-FDG uptake. When uptake characteristics were further stratified, 81.3% of patients showing a *focal spot* myocardial ^18^F-FDG uptake pattern had abnormal MPI findings (*p* < 0.001 vs. normal MPI, Figure 2B). In the overall study population, no significant difference in mean ^18^F-FDG uptake, when measured as SUV _mean_ in the left ventricle, was detected—regardless of the region (septum, apex, lateral wall, or anterior wall; Supplementary Material, Figure S2). Remarkably, patients free of myocardial abnormalities (ischemia, scar, impaired LVEF) rarely displayed focal tracer uptake (Figure 3A–D). Blood glucose levels were similar among the ^18^F-FDG uptake pattern groups (*p* = 0.66).

### 3.3. Prognostic Value of Myocardial ^18^F-FDG Uptake in Patients Undergoing Oncologic PET/CT

We performed a univariate logistic regression analysis with abnormal myocardial perfusion/function on SPECT-MPI as the dependent variable. Cardiovascular risk factors, glucose levels prior to ^18^F-FDG-PET/CT, injected ^18^F-FDG-PET/CT dose, and the myocardial ^18^F-FDG uptake pattern were added as independent variables (Supplementary Material, Table S2). At univariate analysis, we identified the following significant predictors of abnormal myocardial perfusion/function: male sex (odds ratio (OR) 2.21, 95% confidence interval (CI) 1.29–3.97 CI, *p* = 0.004), glucose level prior to ^18^F-FDG-PET/CT (OR 1.17, 95% CI 1.04–1.35 CI, *p* = 0.03), no myocardial ^18^F-FDG uptake (OR 0.52, 95% CI 0.32–0.85 CI, *p* = 0.01), focal and focal spot myocardial ^18^F-FDG uptake (OR 5.1, 95% CI 2.00–13.3 CI, *p* = 0.001 and OR 7.12, 95% CI 1.99–25.63 CI, *p* = 0.003, respectively). At multivariate stepwise logistic regression, focal myocardial ^18^F-FDG uptake remained as a significant predictor of abnormal myocardial function/perfusion (OR 4.72, 95% CI 1.59–14.01, *p* = 0.005, Model 2; and OR 5.32, 95% CI 1.73–16.34, *p* = 0.003, Model 3; Table 3). Further, previous MI and male sex were selected by the model as significant and independent predictors of abnormal myocardial perfusion/function (Table 3). When myocardial abnormalities were subclassified into a reversible perfusion defect (ischemia), irreversible perfusion defect (scar), and impaired LVEF, focal myocardial ^18^F-FDG uptake was an independent predictor of reversible and irreversible perfusion defects (OR 4.17, 95% CI 1.53–11.4, *p* = 0.005 and OR 3.78, 95% CI 1.47–9.69, *p* = 0.006, respectively; Table 4A,B), while the absence of myocardial ^18^F-FDG uptake was selected as a negative predictor of impaired LVEF (OR 0.36, 95% CI 0.19-0.67, *p* = 0.001; Table 4C).

## 4. Discussion

The present study is the first to link focal myocardial ^18^F-FDG uptake pattern in oncologic ^18^F-FDG-PET/CT with abnormal myocardial perfusion and impaired cardiac function. In fact, focal myocardial ^18^F-FDG uptake was found to predict abnormal MPI findings including ongoing myocardial ischemia and myocardial scar by multivariate logistic regression models adjusted for age, sex, body mass index (BMI), blood glucose levels, and other known cardiovascular risk factors. Given the routine use of ^18^F-FDG-PET/CT in oncologic patients and the frequent cardiotoxicity of antineoplastic pharmaceuticals [16,30], our findings imply that patients with focal myocardial ^18^F-FDG uptake on oncologic PET/CT would benefit from further cardiovascular risk assessment.

Our data are consistent with a recent report describing the case of a 60-year-old woman with malignant melanoma in whom focal myocardial ^18^F-FDG uptake was associated with a severe stenosis in the left anterior descending artery (LAD) [13]. The focal ^18^F-FDG uptake was no longer present 5 months following successful LAD-PCI. Similarly, a study encompassing 20 patients with a confirmed focal myocardial ^18^F-FDG uptake pattern on oncologic ^18^F-FDG-PET/CT revealed that focal uptake was associated with a history of CAD [31]. Our study now extends these findings by demonstrating the independent predictive value of the focal myocardial ^18^F-FDG uptake pattern for myocardial ischemia or scar in a large cohort of patients undergoing oncologic ^18^F-FDG-PET/CT.

In current clinical routine, most cancer patients fail to receive adequate cardiovascular follow-up [32]. In fact, the rate of adequately monitored patients was only 36% in a cohort of trastuzumab-treated patients [32]. Biomarkers for an increased risk of impaired LVEF include NT-proBNP, Troponin T, and Troponin I [33]. Although such biomarkers can be integrated in surveillance procedures, they are not specific for cardiovascular abnormalities. Indeed, elevated levels of these biomarkers can be found in various non-cardiac conditions [34], which makes them unsuitable for routine use, particularly in patients with multiple co-morbidities. As such, assessment of myocardial ^18^F-FDG uptake pattern in routine oncologic ^18^F-FDG-PET/CT scanning represents a cost-effective approach that can improve patient monitoring and tailor further cardiovascular work-up to a high-risk population of cancer patients.

Cardiac ^18^F-FDG-PET/CT is routinely applied in clinical practice to assess myocardial viability and inflammation [35,36,37,38,39,40]. Given that the myocardium utilizes free fatty acids (FFAs), glucose, and metabolic intermediates (e.g., lactate, pyruvate, and ketone bodies) as energy substrates [41], the assessment of myocardial viability by ^18^F-FDG-PET/CT requires prior glucose loading to improve image quality and diagnostic accuracy [42]. Thus, myocardial viability assessment is not feasible by oncologic ^18^F-FDG-PET/CT. In contrast, suspected cardiac inflammation (e.g., in sarcoidosis) by ^18^F-FDG-PET/CT is typically investigated in the fasting state, with administration of heparin (or high fat diet) to increase serum free fatty acid (FFA) levels to suppress physiological ^18^F-FDG uptake by cardiac myocytes prior to scanning [36]. Similarly, oncologic ^18^F-FDG-PET/CT preparation protocols aim to reduce physiological myocardial ^18^F-FDG uptake through a minimum fasting duration of 4 h prior to the scan [43]. The latter reduces plasma insulin levels and, thus, glucose uptake into cardiomyocytes [44]. Hence, although significant differences exist in the preparation protocols for inflammation-targeted and oncologic ^18^F-FDG-PET/CT scans, both aim to reduce physiological myocardial ^18^F-FDG uptake. Notably, focal myocardial ^18^F-FDG uptake patterns have also been described in patients with cardiac sarcoidosis [40]. In contrast to oncologic PET, however, patients undergoing inflammation-targeted ^18^F-FDG-PET receive intravenous heparin prior to ^18^F-FDG-PET, which results in an increased sensitivity to detect sarcoidosis-associated lesions [40]. Our study cohort did not include cases of sarcoidosis; however, focal myocardial ^18^F-FDG uptake observed in patients with abnormal perfusion in our study might have resulted from inflammatory cell recruitment to lesion sites. Indeed, early-stage immune responses to myocardial injury have previously been detected by ^18^F-FDG-PET and were associated with reduced functional outcome [12]. Alternatively, a switch from oxidative FFA metabolism to anaerobic glycolysis in ischemic cardiomyocytes might have accounted for the focal ^18^F-FDG uptake pattern observed in our study [45,46].

There are limitations to this study that should be pointed out. First, this study is a single-center retrospective analysis with a limited number of patients that were selected based on the availability of imaging data, which renders the study prone to potential selection bias and therefore limits its generalizability. Second, our study is observational. We report the association between a focal myocardial ^18^F-FDG uptake pattern and impaired cardiac perfusion and function. Our study does not provide information on the underlying mechanism. Third, although a comprehensive group of adjustment variables was employed, unmeasured factors such a*s* serum FFA levels may have affected our endpoints. Fourth, due to the retrospective nature of the study, we did not obtain follow-up data evaluating hard clinical endpoints.

## 5. Conclusions

In summary, our study shows that the myocardial uptake pattern can be used for risk stratification of patients undergoing oncological ^18^F-FDG-PET. In particular, patients with focal myocardial ^18^F-FDG uptake pattern face an increased risk of myocardial impairment and may benefit from further cardiovascular assessment and close monitoring. In times where artificial intelligence and machine learning reshape our healthcare system, advanced pattern recognition algorithms can be used to automate image analysis and identify focal myocardial uptake patterns from whole-body ^18^F-FDG-PET in cancer patients. Exploiting such readily available information represents a powerful and straightforward tool to improve cardiovascular management in oncology.

## Figures and Tables

**Table 1:** Baseline demographics stratified by myocardial ^18^F-FDG uptake pattern.

Baseline Characteristics	Total Cohort n = 302	No Myocardial ^18^F-FDG Uptake n = 109	Diffuse Myocardial ^18^F-FDG Uptake n = 77	Focal Myocardial ^18^F-FDG Uptake n = 24	Focal on Diffuse Myocardial ^18^F-FDG Uptake n = 92	*p*-Value
**Male sex, n (%)**	210 (71.4)	75 (68.8)	50 (64.9)	18 (75.0)	67 (72.8)	0.58
**Age, mean ± SD**	66.8 ± 10.2	66.2 ± 11.1	66.9 ± 9.5	65.1 ± 10.6	68.0 ± 9.7	0.49
**BMI, mean ± SD**	26.2 ± 5.3	26.2 ± 5.1	25.1 ± 5.1	28.6 ± 5.4	26.4 ± 5.3	0.05
**Active malignancy, n (%)**	193 (63.9)	80 (73.4)	43 (55.8)	14 (58.3)	56 (60.9)	0.07
**Diabetes, n (%)**	56 (18.5)	23 (21.1)	8 (10.4)	5 (20.8)	20 (21.7)	0.21
**Hypertension, n (%)**	142 (47.0)	46 (42.2)	40 (51.9)	11 (45.8)	45 (48.9)	0.59
**Smoking, n (%)**	93 (30.9)	35 (32.1)	20 (26.0)	9 (37.5)	29 (31.9)	0.69
**Dyslipidemia, n (%)**	73 (24.2)	25 (22.9)	19 (24.7)	6 (25.0)	23 (25.0)	0.98
**Positive family history, n (%)**	25 (8.3)	9 (8.3)	7 (9.1)	5 (20.8)	4 (4.3)	0.074
**Known CAD, n (%)**	117 (38.7)	45 (41.3)	27 (35.1)	13 (54.2)	32 (34.8)	0.29
**Previous MI, n (%)**	54 (18.9)	17 (15.6)	16 (20.8)	8 (33.3)	16 (17.4)	0.23
**Previous PCI/CABG, n (%)**	82 (27.2)	32 (29.4)	18 (23.4)	7 (29.2)	25 (27.2)	0.83
**Symptoms, n (%)** **Typical angina** **Atypical angina** **Dyspnea** **None**	30 (9.9) 23 (7.6) 39 (12.9) 210 (69.5)	9 (8.3) 8 (7.3) 18 (16.5) 74 (67.9)	7 (9.1) 2 (2.6) 10 (13.0) 58 (75.3)	6 (25.0) 3 (12.5) 1 (4.2) 14 (58.3)	8 (8.7) 10 (10.9) 10 (10.9) 64 (69.6)	0.114
**Chronic pain, n (%)**	90 (30.1)	33 (31.1)	20 (26.0)	11 (45.8)	26 (28.3)	0.30
**Depression, n (%)**	19 (6.5)	6 (5.8)	8 (10.4)	1 (4.3)	4 (4.5)	0.43
**Medication, n (%)** **Statin** **Betablocker** **ACE inhibitors/ARBs** **Aspirin** **Corticosteroids** **Analgesics**	126 (44.5) 146 (51.6) 156 (55.1) 128 (45.2) 40 (14.2) 135 (47.9)	43 (42.6) 52 (51.5) 48 (47.5) 43 (42.6) 17 (16.8) 51 (50.5)	33 (45.2) 36 (49.3) 46 (63.0) 37 (50.7) 9 (12.3) 40 (54.8)	13 (59.1) 14 (63.6) 16 (72.7) 11 (50.0) 4 (18.2) 10 (45.5)	37 (42.5) 44 (50.6) 46 (52.9) 37 (42.5) 10 (11.6) 34 (39.5)	0.53 0.69 0.07 0.65 0.67 0.25
**Creatinine (µM/L), mean ± SD**	116.3 ± 124.0	102.0 ± 94.1	118.1 ± 126.9	173.0 ± 210.3	117.3 ± 122.0	0.11
**CRP (mg/L), mean ± SD**	24.6 ± 43.5	28.5 ± 47.6	19.5 ± 39.0	29.1 ± 37.6	22.8 ± 43.4	0.59
WCB count (10*/µ/L), mean ± SD	7.8 ± 3.3	8.2 ± 3.3	7.3 ± 3.2	8.0 ± 4.4	7.8 ± 2.9	0.44
**NT proBNP (ƞg/L), mean ± SD**	3604.2 ± 13345.4	903.0 ± 1406.3	13584.3 ± 27932.0	2306.2 ± 2229.4	718.4 ± 808.6	0.045

**Table 2:** Baseline demographics stratified by myocardial perfusion/function findings.

Baseline Characteristics	Total Cohort n = 302	Normal Myocardial Perfusion/Function n = 181	Abnormal Myocardial Perfusion/Function n = 121	*p*-Value
**Male sex, n (%)**	210 (71.4)	113 (38.4)	97 (33.0)	0.002
**Age, mean ± SD**	66.8 ± 10.2	66.5 ± 10.5	67.4 ± 9.8	0.45
**BMI, mean ± SD**	26.2 ± 5.3	26.1 ± 5.2	26.3 ± 5.3	0.74
**Active malignancy, n (%)**	193 (63.9)	118 (39.1)	75 (24.8)	0.33
**Diabetes, n (%)**	56 (18.5)	33 (10.9)	23 (7.6)	0.49
**Hypertension, n (%)**	142 (47.0)	84 (27.8)	58 (19.2)	0.44
**Smoking, n (%)**	93 (30.9)	54 (17.9)	39 (13.0)	0.39
**Dyslipidemia, n (%)**	73 (24.2)	42 (13.9)	31 (10.3)	0.36
**Positive family history, n (%)**	25 (8.3)	15 (5.0)	10 (3.3)	0.59
**Known CAD, n (%)**	117 (38.7)	51 (16.9)	66 (21.9)	<0.001
**Previous MI, n (%)**	54 (18.9)	15 (5.0)	42 (13.9)	<0.001
**Previous PCI/CABG, n (%)**	82 (27.2)	34 (11.3)	48 (15.9)	<0.001
**Chronic pain, n (%)**	90 (30.1)	51 (17.1)	39 (13.0)	0.27
**Depression, n (%)**	19 (6.5)	11 (3.8)	8 (2.7)	0.49
**CRP (mg/L), mean ± SD**	24.6 ± 43.5	27.3 ± 51.6	26.3 ± 44.4	0.89
WCB count (10*/µ/L), mean ± SD	7.8 ± 3.3	7.8 ± 2.7	7.8 ± 3.3	0.94
**NT proBNP (ƞg/L), mean ± SD**	3604 ± 13,345	612 ± 946	6956 ± 19,045	0.08

**Table 3:** Predictive value of ^18^F-FDG myocardial uptake pattern for abnormal MPI findings.

Stepwise Logistic Regression Model for Abnormal Myocardial Function/Perfusion in Total Cohort (n = 302)			
Independent Variables	OR	OR (95% CI)	*p*-Value
**Model 1** **Previous MI**	6.50	3.11–13.58	<0.001
**Model 2** **Focal myocardial ^18^F-FDG uptake** **Previous MI**	4.72 6.37	1.59–14.01 3.02–13.44	0.005 <0.001
**Model 3** **Male sex** **Focal myocardial ^18^F-FDG uptake** **Previous MI**	2.15 5.32 6.27	1.13–4.08 1.73–16.34 2.94–13.38	0.020 0.003 <0.001

Stepwise method was performed among age, sex, BMI, injected ^18^F-FDG dose, blood glucose level, no myocardial ^18^F-FDG uptake, focal myocardial ^18^F-FDG uptake, focal spot myocardial ^18^F-FDG, known CAD, previous MI, and previous PCI/CABG. BMI, body mass index; CAD, coronary artery disease; CI, confidence interval; CABG, coronary artery bypass grafting; MI, myocardial infarction; OR, odds ratio; PCI, percutaneous coronary intervention.

**Table 4:** Predictive value of ^18^F-FDG myocardial uptake pattern for ongoing ischemia, myocardial scar, and left ventricular dysfunction.

A. Stepwise Logistic Regression Model for Reversible Perfusion Defect in Total Cohort (n = 302) *			
Independent Variables	OR	OR (95% CI)	*p*-Value
**Model 1** **Focal myocardial ^18^F-FDG uptake**	4.17	1.53–11.4	0.005
B. Stepwise logistic regression model for irreversible perfusion defect in total cohort (n = 302) *			
**Independent variables**	**OR**	**OR (95% CI)**	***p*-value**
**Model 1** **Focal myocardial ^18^F-FDG uptake**	3.78	1.47–9.69	0.006
C. Stepwise logistic regression model for LVEF <50% in total cohort (n = 302) *			
**Independent variables**	**OR**	**OR (95% CI)**	***p*-value**
**Model 1** **No myocardial ^18^F-FDG uptake**	0.36	0.19–0.67	0.001

* Stepwise method was performed among age, sex, BMI, injected ^18^F-FDG dose, blood glucose level, no myocardial ^18^F-FDG uptake, focal myocardial ^18^F-FDG uptake, focal spot myocardial ^18^F-FDG uptake. Only variables staying in the final model are presented. BMI, body mass index; CI, confidence interval; OR, odds ratio.

## Supplementary Materials

The following are available online at https://www.mdpi.com/2077-0383/9/7/2279/s1, Figure S1. Underlying diseases of patients undergoing ^18^F-FDG-PET/CT, Figure S2. Left ventricular ^18^F-FDG-PET/CT uptake given as mean standardized uptake values (SUV_mean_) in different regions of the myocardium (stratified by MPI normal/abnormal findings). No differences were observed in SUV_mean_ across different regions with respect to perfusion findings, Table S1. Baseline ^18^F-FDG-PET/CT and MPI parameters, Table S2. Predictive value of ^18^F-FDG myocardial uptake pattern for abnormal MPI findings.
